# Influence of resin cement shade on the color and translucency of ceramic veneers

**DOI:** 10.1590/1678-775720150550

**Published:** 2016

**Authors:** Daiana Kelly Lopes HERNANDES, Cesar Augusto Galvão ARRAIS, Erick de LIMA, Paulo Francisco CESAR, José Augusto RODRIGUES

**Affiliations:** 1 - Universidade de Guarulhos, Departamento de Odontologia Restauradora, Guarulhos, SP, Brasil.; 2 - Universidade Estadual de Ponta Grossa, Departamento de Dentística, Ponta Grossa, PR, Brasil.; 3 - Universidade de São Paulo, Faculdade de Odontologia, Departamento de Materiais Odontológicos, São Paulo, SP, Brasil.

**Keywords:** Resin cements, Ceramics, Color, Dental veneers

## Abstract

**Objective:**

This *in vitro* study evaluated the effect of two different shades of resin cement (RC- A1 and A3) layer on color change, translucency parameter (TP), and chroma of low (LT) and high (HT) translucent reinforced lithium disilicate ceramic laminates.

**Material and Methods:**

One dual-cured RC (Variolink II, A1- and A3-shade, Ivoclar Vivadent) was applied to 1-mm thick ceramic discs to create thin RC films (100 µm thick) under the ceramics. The RC was exposed to light from a LED curing unit. Color change (ΔE) of ceramic discs was measured according to CIEL*a*b* system with a standard illuminant D65 in reflectance mode in a spectrophotometer, operating in the light range of 360-740 nm, equipped with an integrating sphere. The color difference between black (B) and white (W) background readings was used for TP analysis, while chroma was calculated by the formula C^*^_ab_=(a^*2^+b^*2^)^½^. ΔE of 3.3 was set as the threshold of clinically unacceptable. The results were evaluated by two-way ANOVA followed by Tukey's *post hoc* test.

**Results:**

HT ceramics showed higher ΔE and higher TP than LT ceramics. A3-shade RC promoted higher ΔE than A1-shade cement, regardless of the ceramic translucency. No significant difference in TP was noted between ceramic discs with A1- and those with A3-shade cement. Ceramic with underlying RC showed lower TP than discs without RC. HT ceramics showed lower chroma than LT ceramics, regardless of the resin cement shade. The presence of A3-shade RC resulted in higher chroma than the presence of A1-shade RC.

**Conclusions:**

Darker underlying RC layer promoted more pronounced changes in ceramic translucency, chroma, and shade of high translucent ceramic veneers. These differences may not be clinically differentiable.

## INTRODUCTION

The desire for a beautiful smile has increased, and porcelain veneers have become a valuable treatment for patients seeking better esthetics in anterior teeth. Fractured, discolored or slightly misaligned teeth can be successfully improved with ceramic veneers^1^. However, ceramic material selection for this type of treatment should be carefully done, since the final aesthetic outcome will depend on porcelain contains, which is different in function of brand, and also on thickness, color, translucency, opalescence, fluorescence, surface texture, and shape. Also, the thickness of the underlying resin luting agent plays an important role^1,4,5,11,27^.

Ceramic laminate veneers thickness range from 0.5 to 1.0 mm, therefore, minimally invasive preparation is required^11^. However, previous studies demonstrated that ceramic thickness should be at least 2.0 mm to avoid any substrate influence on the final restoration shade^13,22,26^. Given the reduced thickness of laminate ceramic veneers, its final color is expected to be significantly influenced by dentin discoloration caused by pigment incorporation, trauma, or tetracycline stains. Moreover, shade selection is a complex challenge during porcelain veneer manufacturing, as clinicians usually select ceramic shade based solely on the shade of adjacent teeth, when in fact the final color of the restoration complex depends on the combination of factors such as ceramic restoration underlying tooth structure and resin cement (RC) layer^1,9^.

Most studies evaluating the effect of the cementation on the final restoration shade demonstrated that the final restoration shade depending on the thickness and shade of ceramic veneers and luting agents to match to what the clinician was expecting in the first ceramic shade selection^1,3,8,9,11,13,19^. The significant influence of the thin RC layer on the final aesthetic result has led to the development of resin luting agents with different shades to allow clinicians to select the proper cement shade for laminate veneers to enhance final color match^1^. Nevertheless, other studies demonstrated that RCs have no significant effects on the final color of lithium disilicate glass-ceramics^2,26^. Therefore, the effects of the underlying RC on the final shade of a thin ceramic restoration remains controversial, since it seems to vary according to the ceramic material and RC used^9,11^.

Besides hue, value, and chroma, the total transmittance of a ceramic restoration is a key factor for the final aesthetic outcome and needs to be carefully considered during material selection^16^. Although total transmittance of porcelain veneers is relatively high, especially if compared with porcelain-fused-to-metal restorations, manufacturers have recently developed ceramic systems with varying transmittance (also called high and low translucency materials) in order to offer a better range of light transmission for different clinical situations.

The total transmittance of a ceramic material depends on the absorption and scattering of the incident light. Thus, if most of the light passing through the ceramic is intensely scattered or diffusely reflected, the transmittance will be low and consequently the material will have an opaque appearance. On the other hand, if only part of the light is scattered and the majority is transmitted (high transmittance), the material appears translucent. It is plausible to assume that the final color of highly translucent restorations is more influenced by the shade of the RC than opaque ceramic layers. For all these reasons, the variation in the translucency of all ceramic restorations adds another level of complexity to the color matching process, although little information is available in the literature regarding the effects of the underlying RC on the color and translucency of the ceramic veneer, such as transmittance, shade, and chroma, of highly translucent ceramics.

The purpose of this *in vitro* study was to evaluate the influence of two different shades of resin cement (A1 and A3) layers on the color difference, translucency, and chroma parameters of lithium disilicate ceramic veneers with either high (HT) or low translucency (LT). The null hypotheses were that: a) the color difference promoted by the RC under the HT ceramics is not different from that observed in LT ceramics, regardless of the cement shade; b) there is no difference in the translucency and chroma parameters between HT and LT ceramics when a RC layer is placed under the ceramic plate.

## MATERIAL AND METHODS

### Specimen preparation

To simulate laboratory-processed laminated veneering ceramic restorations, five disk-shaped specimens were fabricated according to the manufacturer’s instructions using lost-wax and heat-pressed techniques (IPS Empress EP 600 press furnace, Ivoclar Vivadent, Schaan, Liechtenstein) for two shades of a lithium disilicate glass-ceramic material (IPS e.max Press HT and LT, A2 shade, n=5/each; Ivoclar Vivadent, Schaan, Liechtenstein). All samples were produced in custom-made rubber molds with 15 mm in diameter and 1.0 mm thick.

Specimens were finished with 400-grit waterproof silicon carbide abrasive papers under running water until the expected thickness was confirmed with a digital micrometer (Ultra-Cal Mark III, Fowler-High Precision Tools & Measuring Instruments, Newton, Massachusetts, USA). Subsequently, all specimens were cleaned in an ultrasonic bath (Vita-Sonic II, VITA Zahnfabrik H. Rauter GmbH & Co, Bad Säckingen, German) for 5 min and no additional treatment or adhesive procedures were made.

**Figure 1:**
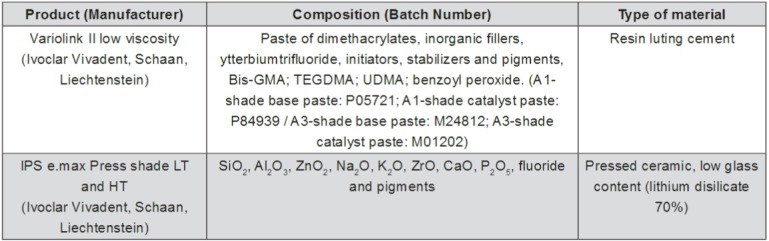
Brand, composition, and batch number of the materials

Abbreviations: TEGDMA: triethylene glycol dimethacilate; Bis GMA: bisphenol A diglycidyl ether methacrylate; UDMA: urethane dimethacrylate

**Figure 2:**
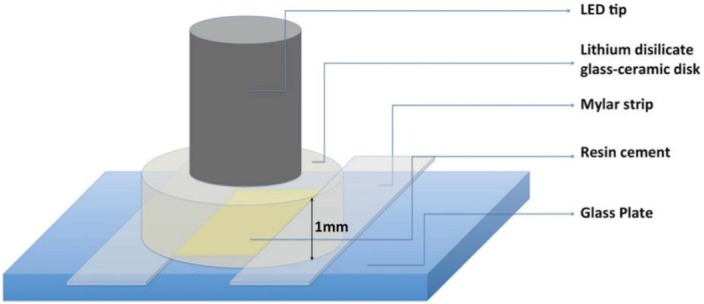
Experimental set up

A Mylar strip was positioned over a glass plate and two adhesive tape strips (3M) were placed over the Mylar strip to act as spacer, ensuring standard thickness for all cements. The RC Variolink II (Figure 1), Base (shades A1 or A3; Ivoclar Vivadent, Schaan, Liechtenstein), and catalyst (A1 or A3, respectively; Ivoclar Vivadent, Schaan, Liechtenstein) were mixed and applied to the glass plate, and the disk-shaped specimen was placed over them to create a RC layer with approximately 100 µm thick underneath the ceramic disc (Figure 2). The samples were photo-activated through ceramic with a LED source (Radii Plus; 2000 mW/cm^2^, SDI Limited, Bayswater, Victoria, Australia). The LED was applied for one minute to ensure polymerization of both cement shades.

### Determination of color and translucency

The color of discs was measured, before and after RCs placement, according to the CIEL*a*b* system, with a standard illuminant D65 in the reflectance in a spectrophotometer (DM-3700d, Konica Minolta Inc., Chiyoda-ku, Tokyo, Japan), operating in the length range of light spectrum λ=360-740 nm, equipped with an integrating sphere. The color of each experimental group was measured over different backgrounds as described below in three coordinate dimensions of L* [from 0 [black] to 100 [white]), a* green-red (−a*=green; +a*=red), and b* blue-yellow (−b*=blue; +b*=yellow). The value of ΔE was calculated according to:

ΔE=[(L^*^_1_−L^*^_0_)^2^+(a^*^_1_−a^*^_0_)^2^+(b^*^_1_−b^*^_0_)^2^]^½^

in which the subscripted 0 represents the color measured in the reflectance mode of the ceramic without cement (control) and the subscripted 1 represents the ceramic color with the respective underlying cement. Both measurements were performed using white background (standard calibration tile with CIE L*=92.95, a*=-0.78, b*=3.57L). Under uncontrolled clinical conditions, average color differences below 3.3 are unnoticeable, due to match in the oral environment. Then, to determine the effect of the resin cements, a ΔE of 3.3 was selected as the clinically unacceptable threshold in this study^11,12,15,17,18,20^.

The translucency parameter (TP) of each specimen was determined by calculating the difference in color between readings over black (standard calibration tile with CIE L*=24.58, a*=0.27, b*=2.58) and white (same tile used for ΔE) backgrounds. TP was calculated according to:

TP= [(L^*^_B_–L^*^_W_)^2^ + (a^*^_B_–a^*^_W_)^2^+(b^*^_B_–b^*^_W_)^2^]^½^

in which the subscripts B and W refer to measurements made on black and white backgrounds. TP is defined as the color difference of a material of a given thickness over white and black backgrounds, and corresponds directly to regular visual assessments. A TP value of zero corresponds to a completely opaque material and the greater the TP value, the higher the actual translucency of the material^11,14^. In addition, the chroma parameter of all groups was calculated using the following formula^27^: C^*^_ab_=(a^*^_2_+b^*^_2_)^½^.

### Statistical analysis

The two-way ANOVA in a multivariate general linear model was used to detect significant differences between groups, with the factors “ceramic translucency”, “cement shade” and the interaction between them, followed by Tukey’s *post-hoc* test at a pre-set alpha of 5%. *Post-hoc* power test was performed for both factors. The data were analyzed by a personal statistical software (Statistics 19, SPSS Inc, IBM Company, Armonk, New York, USA).

## RESULTS

Two-way ANOVA showed statistically significant differences in ΔE, TP, and chroma values for the factors ceramic translucency and cement shade (p<0.0001) with power higher than 90%, but no statistically significant interaction between factors was detected (p>0.05) for any of the evaluated parameters. The ΔE values were within clinically acceptable limits (ΔE<3.3). The ΔE value obtained for HT ceramic was significantly higher than that of LT ceramic, regardless of RC shade. Also, the ΔE was significantly higher when A3-shade RC was used than when A1-shade cement was placed under the ceramics, regardless of ceramic translucency (Table 1).

**Table 1:** Color difference (ΔE) in LT and HT ceramics after cement insertion (standard deviations), the means followed by different letters [upper case letters within the columns (ceramic) and lower case letters within the rows (cement)] are significantly different (p<0.05)

Cement shade	LT	HT	Average of each cement
A1	1.46 (0.35)	1.93 (0.31)	1.69 (0.40)^a^
A3	2.55 (0.37)	3.04 (0.27)	2.79 (0.40)^b^
Average of each ceramic	2.00 (0.67)^A^	2.49 (0.65)^B^	

The TP of HT ceramic was significantly higher than that of LT ceramics, regardless of the presence of RC. No significant difference in TP was noted between ceramic discs with A1-shade RC and those with A3-shade cement. However, TP was significantly lower in ceramic discs with underlying RC layer in comparison to that observed in ceramic discs without RC (Table 2). On the other hand, the chroma of HT ceramic was significantly lower than that of LT ceramic, regardless of the RC shade. There was no significant difference between the chroma of ceramics with A1-shade RC and that of ceramics without an underlying RC layer. However, the presence of A3-shade RC layer resulted in higher chroma than that obtained in the presence of A1-shade RC layer or without underlying RC (Table 3).

**Table 2:** Translucency parameter (TP) means (standard deviations) of LT and HT ceramics before and after cement insertion. The means followed by different letters [upper case letters within column (ceramic) and lower case letters within row (cement)] are significantly different (p<0.05)

Cement shade	LT	HT	Average of each cement
No cement	17.04 (0.48)	17.87 (0.46)	17.46 (1.12)^b^
A1	15.42 (0.27)	16.14 (0.42)	15.78 (0.75)^a^
A3	15.34 (0.56)	16.46 (0.77)	15.90 (0.92)^a^
Average of each ceramic	15.93 (0.91)^A^	16.82 (0.94)^B^	

**Table 3:** Mean of C*ab (standard deviations) of LT and HT ceramics before and after placement of RC with different shades. The means followed by different letters [upper case letters within the columns (ceramic) and lower case letters within the rows (cement)] are significantly different (p<0.05)

Cement shade	LT	HT	Average of each cement
No cement	21.44 (1.25)	17.47 (0.13)	19.46 (2.25)^b^
A1	21.62 (0.99)	18.97 (0.98)	19.85 (1.98)^b^
A3	22.73 (1.20)	19.86 (0.36)	21.29 (1.73)^a^
Average of each ceramic	21.93 (1.22)^A^	18.47 (1.08)^B^	

## DISCUSSION

In this study, the ΔE values measured for HT ceramics were higher than those observed in LT ceramics. Also, the use of A3-shade RC under the ceramics resulted in higher ΔE than the use of A1-shade RC, regardless of ceramic translucency. Therefore, the first alternative hypothesis stating that the change in color promoted by the RC under the HT ceramics is not different from that observed in LT ceramics regardless of the cement shade was rejected. These findings are in agreement with other studies and confirm the evidences that ceramic restorations with thicknesses lower than 2.0 mm are susceptible to color changes depending on the RC shade^10,13,24,25^. In agreement with our results, Chen, et al.^9^ (2015) observed that resin cements can affect the final color of ceramic veneer restorations (IPS e.Max Press, LT A3) and the extent of this effect varies according to the resin cement shades. However, it should be pointed out that the lithium disilicate glass ceramics evaluated in this study are more translucent than other ceramic types (e.g. polycrystalline and glass-infiltrated composites)^3,11^, therefore it is expected that the effects of underlying RC layer on the restoration shade should be less pronounced in other ceramics. Çömlekoğlu, et al.^11^ (2015) observed in a multilayered glass-ceramic veneers that color changes for body and cervical regions were not affected by resin color or ceramic thickness, but the incisal area was affected.

The higher translucency of lithium disilicate ceramics is a consequence of its unique microstructure containing large amount of a glassy phase and a relatively translucent crystal (lithium disilicate, Li_2_Si_2_O_5_)^1^.

The presence of a RC layer under LT ceramics led to an average color change of 1.46 and 2.55 for A1- and A3-shade RC layers, respectively. On the other hand, for the HT ceramics, the presence of A1- and A3-shade RC layers under the ceramics promoted an average color change of 1.93 and 3.04, respectively. The clinical impact of such changes in color remains controversial. Some studies have shown that color differences greater than one unit (ΔE*ab>1) can be visually perceived only by 50% of human observers^7,21,23^, while others demonstrated that the population in general distinguishes differences in color only when ΔE*ab values are higher than 3.3^12,20^. On the other hand, Seghi, et al.^21^ (1989) noted that color differences with ∆E threshold of 2.0 in monochromatic porcelain specimens may be correctly judged by 100% of observers. Also, Paris, et al.^18^ (2013) used the threshold ∆E<3.7 to consider their results not clinically differentiable. Therefore, as the color changes observed in this study are within this threshold, the differences caused by cements could be detected by a human eye but could still be clinically acceptable^15,17,18,20^.

Besides the change in color observed in the evaluated ceramics with an underlying RC layer, significant changes in ceramic TP and chroma were also observed, so the second alternative hypothesis was rejected. More specifically, the presence of a RC layer under the ceramic laminate did decrease ceramic translucency, while the use of A3-shade RC under both ceramics increased the chroma. This finding indicates that the presence of a RC layer under the ceramic veneer may help mask the color of the underlying tooth substrate. However, it should be pointed out that, regardless of the presence of cement, HT ceramics always exhibited higher translucency than LT ceramics. Indeed, HT ceramics showed the closest TP values (17.87) to human enamel (18.7), as measured in a study that evaluated tooth substrates under the same conditions as in this study (1-mm thick specimens)^9^. Therefore, it is important for clinicians to be able to predict the translucency of ceramic laminates when the RC is placed under them, instead of relying solely on the original translucency of such products.

Color changes were measured in ideal conditions by a spectrophotometer, so the small differences observed could be barely detected by the human eye^27^. Three dimensional objects produce a two-dimensional image via perspective projection on the retina^6^. The image varies with distance from the center of the projection. Even when equated for size, the nose looks relatively larger and the ears smaller as the distance decreases. However, it is worth noting that, along with the change in ceramic shade observed in here, we also observed significant changes in ceramic translucency and chroma. Therefore, it is reasonable to assume that all those changes together may result in perceptible and unacceptable differences by the human eye in comparison to the color and translucency of the adjacent teeth. Only further studies evaluating a simulated clinical situation comparing restored teeth with adjacent sound teeth would confirm such speculation.

In this study, only one ceramic shade (A2) and one ceramic type with variable translucency were evaluated, so the results cannot be extrapolated to other ceramic types and shades. Moreover, although the use of cement base paste solely rather than the mixture between catalyst and base pastes is recommended for the cementation of ceramic veneers, the base and catalyst pastes of the RC were mixed and applied to the ceramic disc. This was done to ensure that the best polymerization was achieved in all specimens, as it can affect the resin shade^28^.

Based on the current results, to achieve optimal aesthetic results, clinicians must be aware not only of the color and translucency of ceramics, but also of the effects of the underlying RC layer on their optical properties, since other cement shades and ceramic combinations could produced clinically unacceptable color change^10^. Considering that this study demonstrated that the use of different cement shades may change the color, translucency, and chroma of ceramic laminates, care should be taken when selecting the RC shade. This issue deserves more attention when high translucent ceramic laminates are associated with darker shade RCs.

## CONCLUSION

Within the limits of this study, it can be concluded that since HT ceramic are more translucent than LT ceramic, the use of RCs may change their final ceramic translucency, chroma, and shade. Although some of these differences may not be clinically differentiable, care must be taken in the selection of cement shade to achieve the best final results possible.
